# Fatal Free Falls: A Clinical and Forensic Analysis of Skeletal Injury Patterns Using PMCT and Autopsy

**DOI:** 10.3390/jcm14227912

**Published:** 2025-11-07

**Authors:** Filip Woliński, Jolanta Sado, Kacper Kraśnik, Justyna Sagan, Łukasz Bryliński, Katarzyna Brylińska, Grzegorz Teresiński, Tomasz Cywka, Marcin Prządka, Robert Karpiński, Jacek Baj

**Affiliations:** 1 Department of Forensic Medicine, Medical University of Lublin, Jaczewskiego 8b, 20-090 Lublin, Poland; 2 Department of Correct, Clinical, and Imaging Anatomy, Medical University of Lublin, Jaczewskiego 4, 20-090 Lublin, Poland; 3 Department of Orthopedics and Movement Traumatology, Provincial Integrated Hospital, Szpitalna 45, 62-504 Konin, Poland; 4 Institute of Medical Sciences, Faculty of Medicine, The John Paul II Catholic University of Lublin, Konstantynów 1H, 20-708 Lublin, Poland; 5 Department of Machine Design and Mechatronics, Faculty of Mechanical Engineering, Lublin University of Technology, Nadbystrzycka 36, 20-618 Lublin, Poland

**Keywords:** fatal free falls, forensic pathology, skeletal trauma, postmortem computed tomography (PMCT), autopsy, injury patterns, suicide vs. accident, blunt force trauma, pelvic and vertebral fractures, medico-legal analysis

## Abstract

**Background:** Free fatal falls (FFF) are a frequent occurrence in forensic medicine. Many variables, such as the victim’s sex, BMI, intoxication, height of the fall, and mental illness, can influence injury patterns. Previous studies identified fracture patterns and frequencies mostly with general anatomical detail, focusing on broad areas. As specific fractures might be roots for new statistical connections, this leaves a gap in our understanding. Using postmortem computed tomography, we aim to establish fracture frequencies and identify possible new statistical connections. **Methods:** In total, we retrospectively analyzed seventy-nine cases of confirmed deaths due to falls using the database of the Department and Institute of Forensic Medicine in Lublin. Our inclusion criteria were death due to free fall onto hard, non-deformable surfaces. We excluded cases of ground-level falls. All victims must have undergone postmortem computed tomography. Furthermore, each analyzed case documented individual intrinsic variables (sex, age, body mass, height, pre-existing mental conditions, and drug or alcohol use) and extrinsic variables (fall height, landing surface, time between the fall and death, and known cause of the fall). **Results**: Injuries in free fatal falls tend to focus on the axial skeleton. Suicides experience more severe, bilateral fractures, often involving the pelvis and limbs, while accidents tend to have unilateral injuries with rare limb involvement. We established new correlations with the height of the fall for the maxilla, mandible, anterior and posterior regions of the occipital bone, and the temporal bone. Moreover, our research confirmed previously noted correlations between the height of the fall and fractures of the limbs (and their individual bones), the lumbar vertebrae, and the chest. **Conclusions:** Our findings highlight that free fatal falls are characterized by distinct skeletal injury patterns that differ between accidents and suicides, with bilateral pelvic and limb fractures being particularly indicative of intentional falls. The integration of PMCT with autopsy improves the detection of these patterns. It provides valuable diagnostic and medico-legal insights, supporting a more precise determination of the cause and manner of death.

## 1. Introduction

Falls from height are classified as the second most common cause of blunt trauma, following motor vehicle accidents [1]. They can be fatal and lead to violent death [2,3]. Fatal free falls (FFF) result from suicide, homicide, or accident [4] with work-related incidents and intentional acts being the most common. These events account for a significant proportion of traumatic fatal injuries globally, comprising 23% to 56% of intentional deaths and 35% to 37% of construction work-related deaths [5]. FFFs occur more frequently in urban settings, with balconies, buildings, and trees being the most common locations. Some studies have focused on analyzing the characteristics of individuals statistically associated with a higher occurrence of such incidents. These analyses indicate that men are most often affected, likely because they are more often involved in jobs that require work at significant heights than women. In contrast, women account for a higher proportion of suicide-related FFFs than men. The average age of individuals involved in these incidents varies considerably across studies, highlighting that people of all ages are at risk of FFFs [6,7].

Due to the numerous factors involved, the consequences of falls can vary. Outcomes depend on body mass, fall height, surface type, secondary impacts, point of first contact, and age-related comorbidities. The manner of the fall—whether intentional or accidental—also plays a crucial role [8,9]. Suicides tend to land on their feet, while accidental fallers reportedly have a higher frequency of skull and upper extremities fractures [10]. Despite these differences, it has been observed that, in general, victims of FFF most commonly demonstrate fractures of the skull, vertebrae, and thoracic cavity, followed by fractures of the lower extremities, upper extremities, and pelvis [11]. This suggests that victims of FFF show specific patterns of skeletal blunt force trauma. Such a distribution of injuries results from the unique force interactions involved [12].

FFF is associated with high-energy vertical deceleration [13]. Literature indicates that injury patterns are related to fall height and the kinetic energy at impact, as the body undergoes rapid deceleration upon striking the ground [14]. During this process, kinetic energy is transferred to the ground and exerted back onto the body, resulting in injuries [15]. Significant correlations have been established between the height of the fall, the energy imparted to the body during BFT, and the distribution and extent of skeletal injuries [16].

Osseous injuries play a significant role in forensic pathology. These fractures can be identified using post-mortem computed tomography (PMCT) [17], external body examination, and forensic autopsy. Forensic pathologists investigating victims of falls from height are required to evaluate injuries, establish correlations between injury severity and fall height, and assess the medico-legal aspects of the incident. Providing such information can be particularly challenging in several scenarios. First, in the absence of eyewitness accounts, determining whether a fall from height resulted from homicide, suicide, or an accident becomes more difficult. Another challenge arises from ambiguous injuries, which could suggest either a fall from height or other causes of violent death, such as motor vehicle accidents [18,19]. PMCT may play a significant role in addressing these difficulties. It serves as an essential complement to conventional autopsy in trauma victims, facilitating the detection of numerous additional injuries. PMCT has proven useful across a broad range of forensic applications, including the assessment of blunt-force injuries, firearm and sharp-force trauma, blast injuries, asphyxic injuries, pediatric deaths, and archaeological/anthropological examinations [20,21,22]. However, there is a limited number of studies that include analyses of PMCT in victims of FFF and report fracture frequencies with anatomical detail. As that information might be the root of new correlations, this leaves gaps in our understanding of FFF.

Furthermore, complex pelvic injuries in fatal falls might be underreported in autopsy studies because the pelvis is not typically dissected. Using postmortem computed tomography, we identified complex blunt trauma patterns commonly associated with falls, including faller’s fractures, Bilateral pubic rami fractures, and pelvic injuries classified by the Young–Burgess system.

The faller’s fracture, typically seen in suicide jumpers, results from massive axial loading and presents as a horizontal sacral fracture with bilateral vertical extensions, forming an H-shaped pattern [23]. Bilateral fractures of the superior and inferior pubic rami are often caused by falls in a position where the thighs are abducted [24].

The Young–Burgess classification categorizes pelvic injuries into:

- APC (anterior–posterior compression): progressing from pubic symphysis disruption to sacroiliac injury.
- LC (lateral compression): associated with pubic rami and sacral ala or iliac wing fractures.
- PAC (posterior–anterior compression): associated with faller’s fracture or double vertical fractures of the posterior aspect of the pelvis.
- VS (vertical shear): from axial loading, causing vertical displacement of one hemipelvis and disruption of pelvic ligaments [25,26,27].

This study aims to investigate whether FFF are characterized by a specific pattern of skeletal injuries that distinguishes them from other types of violent deaths. It also sought to determine whether such a pattern enables a reliable assessment of the cause and circumstances of death, thereby contributing to the improvement of postmortem diagnostics. The study includes an analysis of PMCT in FFF victims.

## 2. Materials and Methods

We analyzed 134 cases of fatal falls from 1 January 2016 to 7 September 2024, using the database of the Department and Institute of Forensic Medicine in Lublin, Poland.

Our inclusion criteria were death due to free fall onto hard, non-deformable surfaces. For this study, a free fall was defined as a vertical descent from a height occurring without intermediate obstacles, secondary impacts, or collisions with other structures. Falls involving multiple impacts or any intermediate obstructions (such as tree branches, balconies, or glass surfaces) were excluded from analysis. We excluded all cases of ground-level falls, defined as falls from the standing or sitting position without a vertical drop. The described injuries could not be attributed to any causes other than the fall itself. Furthermore, each analyzed case must have documented individual intrinsic variables (sex, age, body mass, height, pre-existing mental conditions, drug or alcohol use) and extrinsic variables (height of the fall, landing surface, time between the fall and death, and known cause of the fall). Any cases without complete documentation were excluded from the study. Within our department, it is standard procedure for all trauma-related cadavers to undergo PMCT before autopsy; consequently, all cases incorporated into this study had accessible scans. PMCT assessments were performed independently and blinded to autopsy results. Following both examinations, the findings were compared, and any inconsistencies were resolved during a second consensus evaluation of the available data.

Of the 134 cases initially reviewed, 16 were excluded as non–free-fall incidents (e.g., falls on stairs or falls obstructed by glass, furniture, or other objects), 13 as ground-level falls, 10 due to additional trauma sustained before the fall (such as assault or traffic accidents), and 16 because of incomplete documentation. Seventy-nine cases met the inclusion criteria for this study.

Using autopsy reports and PMCT, victims were assessed for skeletal injuries. The Department and Institute of Forensic Medicine in Lublin possess a Toshiba TSX-034A Astelion Advance Edition Computer Tomography system (Canon Medical Systems Corporation, Otawara, Tochigi, Japan) and a dedicated imaging data archiving subsystem (PACS) with scalable disk space (NAS), which provides access to all previously performed examinations. All cases were analyzed for skeletal trauma using the RadiAnt DICOM Viewer program (Version 2025.2, Medixant, 2025), which implements 3D reconstruction, alongside the Viterea diagnostic console certified for clinical diagnostics (MD) with an MD-class EIZO RX440 monitor (EIZO Corporation, Hakusan, Ishikawa, Japan).

This study’s analysis was divided into two sections based on the cause of the fall: suicide or accident. The gathered data were examined for bone fractures, first categorizing them into six main categories: head, spine, thorax, pelvis, upper limbs, and lower limbs. Fractures were further classified by creating additional anatomical subcategories, including individual bones and their respective parts. The study accounted for the height of the fall, dividing it into two classifications. The first classification is based on the literature and comprises two groups: falls from less than 3 m and falls from 3 m or more. The second classification consists of eight groups: falls less than or equal to 3 m, falls from 3 to 6 m, falls from 6 to 10 m, falls from 10 to 15 m, falls from 15 to 20 m, falls from 20 to 25 m, falls from 25 to 30 m, and falls of 30 m or higher. These groups were formed to perform a statistical analysis, as using regular intervals would yield insufficient subject numbers in each height group. In cases where the height of the fall was reported in stories instead of meters, it was assumed that a story is approximately 3 m high (3-story building = ground floor + 3 floors = 3 m + 3 × 3 m =12 m). The study also accounted for the kinetic energy of the fall, calculated from the potential energy using the formula Epot = mgh (m: body mass, g: acceleration due to gravity (9.81 m/s^2^), and h: height in m).

All data were analyzed using Statistica software (StatSoft, version 13), with statistical significance set at *p* ≤ 0.05. Depending on the type of data and its distribution (whether normal or not), various tests were employed, including Student’s *t*-test (T-test), Mann–Whitney U test (MWU), Chi-squared test (χ^2^ test), Spearman’s rank correlation coefficient (ρ), and Kruskal–Wallis test (ANOVA). To assess the influence of the studied variables, we used Binary Logistic Regression.

## 3. Results

Seventy-nine cases of fatal falls from height were analyzed, including 22 women (28%) and 57 men (72%). The average age was 54; the median was 55, including the minimum age of the fatal fall victim—17—and the maximum age of 93. The average BMI was 25.08 kg/m^2^, with a median of 24.11 kg/m^2^, ranging from 15.15 kg/m^2^ to 40.64 kg/m^2^.

Of the victims, 26 (32.91%) were under the influence of alcohol, including four women (18.18%) and 22 men (38.60%). The maximum concentration of alcohol in the victim’s blood was 7.48 g/L. Of the examined cases, mental illness was reported in 20 cases (25.32%), including 11 women (50%) and nine men (15.79%). The remaining 59 cases (74.68%), including 11 women (50%) and 48 men (84.21%), had no mental illness history. A psychiatric history was significantly more prevalent among females than among males (χ^2^ test, *p* = 0.0017). Additionally, individuals with mental illness tended to be under the influence of alcohol (χ^2^ test, *p* = 0.0116).

Of the examined cases of falling from a height, an accident occurred in 36 cases (45.57%), and a suicide occurred in 43 cases (54.43%). Among the deceased, there were 20 low falls (25.32%), including 2 women (9.09%) and 18 men (31.58%). Falls above 3 m were classified as high falls, which accounted for 59 cases (74.68%), including 20 female falls (90.91%) and 39 male falls (68.42%). High falls were divided into specific fall height ranges. The average fall was 22.30 m, the median was 7 m, the minimum fall height was 1.5 m, and the maximum fall height was 1000 m. Women, on average, fell from higher elevations than men (MWU, *p* = 0.0276); however, within the suicide group, men experienced falls from greater heights (MWU, *p* = 0.001).

The time of death varied from death on site- 46 cases (58.23%), including 13 among women (59.09% of surveyed women) and 33 among men (57.89%), to death after more than 10 days from fall—2 cases (2.53%), which were exclusively men (3.51%). The maximum time to death from a fall was 20 days. In one case, the exact time of death from the fall was unrecorded; however, it was known to occur within several hours. The collected data are presented in Table 1.

Accidents

Of the 79 cases of falls from height, 36 were caused by accident (45.57%), including four women (18.18% of all female falls) and 32 men (56.14% of all male falls). The minimum fall height was 1.5 m, and the maximum was 1000 m. The average height of fall was 5.94 m. Considering the presence of a parachutist who fell from a height of 1000 m in the accident, it is not included in the average due to its false inflation. The median was 4 m.

Falls from a low height occurred in 15 cases (41.67% of accident victims), all of whom were men (46.88% of accidents among men). Falls from a high height (3–15 m) occurred in 19 cases (52.78%), including 4 cases involving women (100%) and 15 among men (46.88%). Falls from a height higher than or equal to 15 m concerned 2 cases (5.56% of cases), all of which were exclusively among men (6.25% of men).

Of the accident victims, 17 of them were under the influence of alcohol (47.22% of accident victims), including one woman (25% of women) and 16 men (50% of men). Higher alcohol intoxication was statistically correlated with accidental deaths (χ^2^ test, *p* = 0.0133). Three victims had a documented mental illness (8.33% of accident victims), including one woman (25% women) and two men (6.25% of men). The particulars were presented in the Table 2.

Suicide

Of the 79 cases of fatal falls from height, 43 were caused by suicide (54.43%), including 18 women (81.82% of all female falls) and 25 men (43.86% of all male falls). The minimum fall height for suicide was 3 m, and the maximum was 33 m. The average height of falls was 12.88 m. The median was 9 m. Females were more likely to die by suicide, whereas males were more frequently victims of accidental falls (*p* = 0.0024). Additionally, a significant correlation was observed between fall height and manner of death, with suicides occurring from greater altitudes (MWU, *p* = 0.00013).

Falls from low height occurred in 5 cases (11.63% of suicide victims), including two among women (11.11% of women) and three among men (12% of men). Falls from a high height (3–15 m) occurred in 24 cases (55.81%), including nine among women (50%) and 15 among men (60%). Falls from a height of 15 m or higher were involved in 14 cases (32.56% of cases), including 7 cases among women (38.89% of women) and seven among men (28% of men). Furthermore, we found that suicides tend to have greater kinetic energy (MWU, *p* = 0.0044) and lower BMIs (MWU, *p* = 0.0163) than accidents.

Of the suicide victims, 9 of them were under the influence of alcohol (20.93% of suicide victims), including three among women (16.67% of women) and six among men (24% of men). Seventeen victims had documented mental illness (39.53% of suicide victims), including 10 among women (55.56% of women) and seven among men (28% of men). Psychiatric history had a positive correlation with suicide (χ^2^ test, *p* = 0.0015). The particulars were presented in the Table 3.

Skull

Skull fractures occurred in 47 victims who fell from a height. We observed a bimodal distribution of skull fractures, with peaks at altitudes below 6 m and above 20 m (χ^2^ test, *p* = 0.010). Neurocranial fractures were more common among males (χ^2^ test, *p* = 0.003). Fracture frequencies are presented in Table 4.

Positive correlation was found between maxillary fractures and both the height of the fall (MWU, *p* = 0.01) and its kinetic energy (MWU, *p* = 0.008). Additionally, fractures of the mandible showed positive correlations with the fall height category (MWU, *p* = 0.009) and the fall’s kinetic energy (MWU, *p* = 0.008). Furthermore, logistic regression analysis showed that as skull fractures increase, pelvic fractures decrease by almost 70% (OR = 1.065; 95% CI: 1.01–1.12; LRT: *p* = 0.011).

Accidents vs. suicides

**Figure 1:**
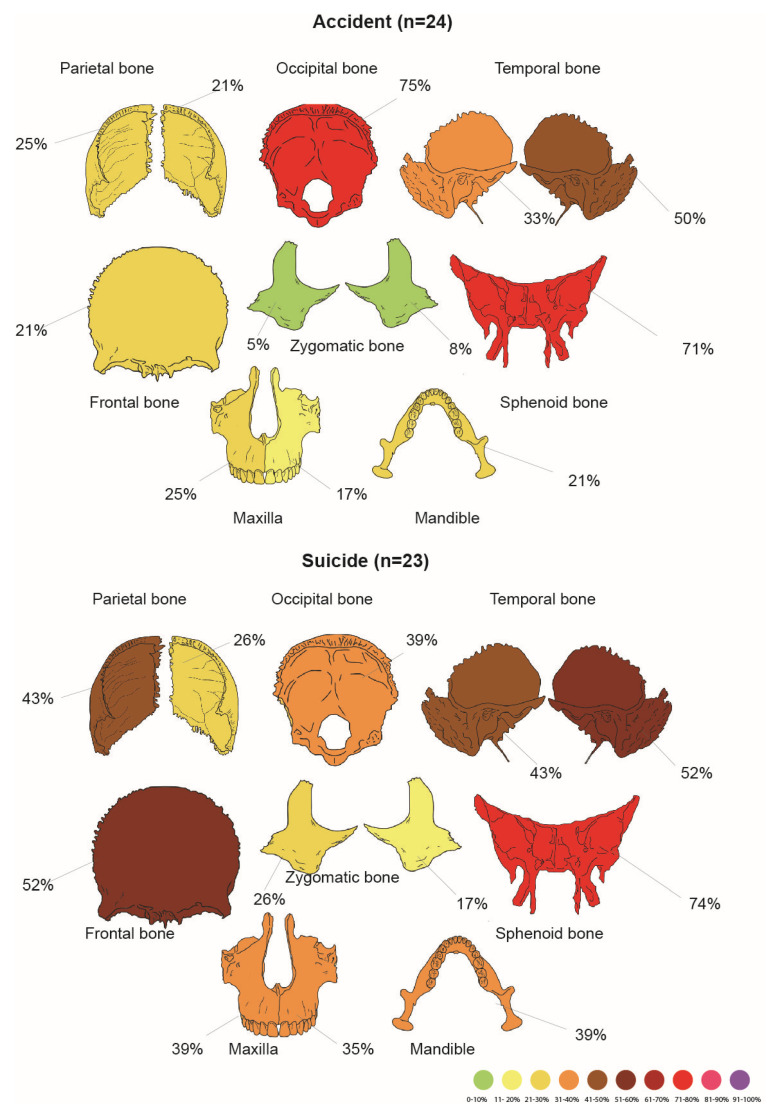
Comparison between main skull fractures in accidental and suicidal cases. For details on the subparts of every bone, refer to the Table 5 and Table 6.

Comparison between injuries to the skull in suicides and accidents is shown in Figure 1.

**Table 5:** Skull fractures in accidental fatal falls.

Damaged Structure	All Cases	% of Cases	Bilateral/Complex Injury
Sphenoid bone	17	68	7—complex injury
Body of the sphenoid bone	10	40	-
Greater wing of the sphenoid bone	12	48	5
Pterygoid process of the sphenoid bone	1	4	1
Temporal bone	15	60	5
Petrous part of the sphenoid bone	9	36	4
Squamous part of the sphenoid bone	11	44	2
Occipital bone	18	72	
Posterior part of the occipital bone	9	36	4—Anterior + posterior part
Anterior part of occipital bone	13	52	
Maxilla	7	28	3
Frontal bone	5	20	-
Parietal bone	8	32	3
Nasal bone	3	12	2
Ethmoid bone	3	12	-
Palatine bone	1	4	0
Vomer	3	12	-
Zygomatic bone	4	16	0
Separation of the zygomatic bone	2	8	1
Zygomatic arch	7	28	1
Mandible	5	20	-
Body of the mandible	4	16	1—Body + Left and Right ramus
Ramus of the mandible	3	12	1

**Table 6:** Skull fractures in the suicide group.

Damaged Structure	All Cases	% of Cases	Bilateral/Complex Injury
Sphenoid bone	17	70.83	8—complex injury
Body of the sphenoid bone	7	29.17	-
Greater wing of the sphenoid bone	14	58.33	4
Pterygoid process of the sphenoid bone	1	4.17	1
Temporal bone	15	62.5	7
Petrous part of the sphenoid bone	11	45.83	6
Squamous part of the sphenoid bone	14	58.33	3
Occipital bone	9	37.5	
Posterior part of the occipital bone	6	25	4—Anterior + posterior part
Anterior part of occipital bone	7	29.17	
Maxilla	12	50	5
Frontal bone	11	45.83	-
Parietal bone	12	50	4
Nasal bone	5	20.83	4
Ethmoid bone	5	20.83	-
Palatine bone	3	12.5	2
Vomer	1	4.17	-
Zygomatic bone	8	33.33	3
Separation of the zygomatic bone	3	12.5	1
Zygomatic arch	12	50	2
Mandible	9	37.5	
Body of the mandible	8	33.33	0—Body + Left and Right ramus
Ramus of the mandible	2	8.33	0

In statistical analysis, the anterior part of the occipital bone was fractured more frequently in suicides than in accidents (χ^2^ test, *p* = 0.043).

Rib cage

Among 79 cases of fatal falls, rib cage injury occurred in 70 cases (88.61% of all cases). The fracture frequencies in the studied population are shown in Table 7.

Rib cage fractures increased with fall height (ANOVA, *p* = 0.00022) across categories of less than 3 m, 6–10 m, 20–25 m, and 25–30 m, with similar trends observed for individual rib fractures. Additionally, higher kinetic energy correlated with a greater total number of chest fractures (ρ, *p* = 0.0002). Males sustained rib cage fractures more frequently than females (MWU, *p* = 0.0066), and individuals with mental illness exhibited more severe rib cage injuries (MWU, *p* = 0.0123).

Accident vs. Suicide

**Figure 2:**
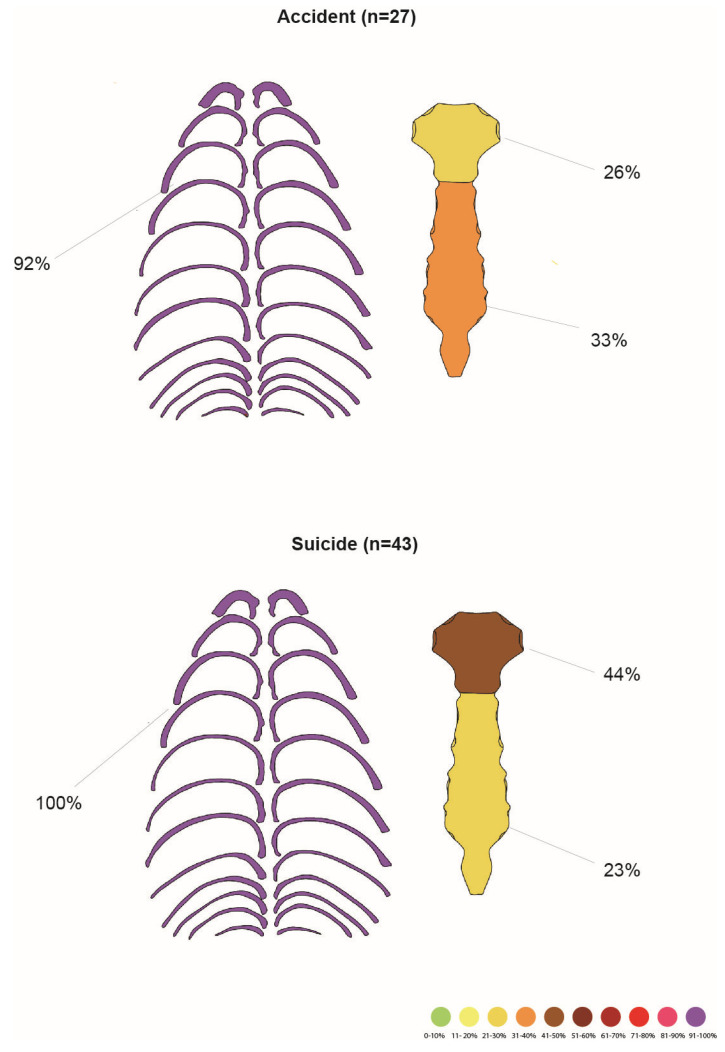
Comparison between rib and sternal fractures of accidental and suicidal FFF. For more information, refer to the tables.

Among 36 victims of the accident, 27 had a rib cage injury (75% of all accident victims). No statistical correlation was found between the height of the fall and rib cage injuries (MWU, *p* = 0.513). Fracture frequencies are presented in Table 8 and Figure 2.

All 43 suicide fall victims suffered chest injuries. Bilateral rib fractures were significantly more frequent in suicides, while unilateral fractures were more prevalent in accidents (χ^2^ test, *p* = 0.0001). Fracture frequencies are presented in Table 9.

Vertebrae:

Among the 79 victims, 61 cases (77.22% of all cases) had a vertebral injury, including 18 women (81.82% of women) and 43 men (72.88% of men). Fracture frequencies are presented in Table 10.

We observed a statistically significant correlation between the height of the fall and the presence of vertebral fractures (MWU, *p* = 0.043). Every meter fallen increased the chance for a spinal fracture by 8.7% (OR = 1.087; 95% CI: 0.99–1.20; LRT: *p* = 0.034). Moreover, the lumbar spine was the only spinal segment to show a correlation with fall height (MWU, *p* = 0.000004). The differences were most pronounced at height groups of less than 3 m, 20–25 m, and over 30 m. The most significant differences were observed between the less than 3 m and the above 30 m categories. Additionally, a relationship was found between vertebral fractures and the kinetic energy of the fall (MWU, *p* = 0.033).

Binary logistic regression revealed a strong and statistically significant association between sternocostal and vertebral fractures, with the odds of vertebral injury increasing nearly tenfold as the number of sternocostal fractures rose (OR = 9.67; 95% CI: 2.12–44.15; LRT *p* = 0.0024).

Accident vs. Suicide

**Figure 3:**
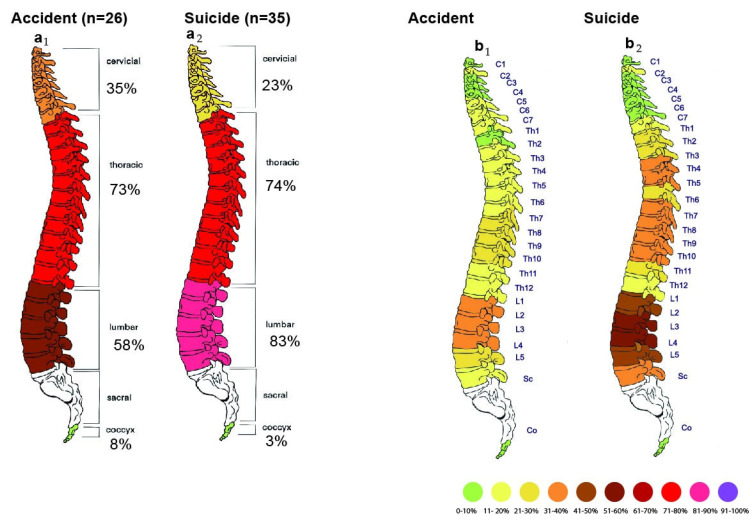
Comparison of accidental and suicidal fractures of the spinal column. (**a_1_**,**a_2_**)—Fractures in the area of the spine, (**b_1_**,**b_2_**)—fractures of individual vertebrae. The most significant differences were observed in the lumbar vertebrae.

Comparison between spinal column injuries in accidents and suicides is shown in Figure 3. 

Among the 36 victims of the accident, 26 suffered spinal injuries (72.22% of all accident victims). Fracture frequencies are presented in Table 11.

Among the 43 victims of a suicidal fall from a height, spinal injuries occurred in 35 cases (81.40% of all suicide victims). Fracture frequencies are presented in Table 12.

Pelvis

Of the 38 individuals with pelvic fractures, a significant correlation was found with female sex (χ^2^ test, *p* = 0.026). Pelvic fracture frequencies are shown in Table 13.

In the statistical analysis, we observed that fractures of the inferior pubic ramus were more likely to occur in individuals with lower BMI than in those without this damage (MWU, *p* = 0.035). Additionally, the base (MWU, *p* = 0.016) and middle part (MWU, *p* = 0.027) of the sacrum were more likely to be affected in cases with a lower BMI. Logistic regression analysis showed that body mass was a significant predictor of pelvic damage, and with every kg of mass, the probability of pelvic fracture decreased 5.4% 17.9% (OR = 0.95; 95% CI: 0.91–0.98; LRT: *p* = 0.0019). Another observation was that fractures of the pubis (χ^2^ test, *p* = 0.025), sacrum (χ^2^ test, *p* = 0.004), and ischium (χ^2^ test, *p* = 0.015) were more common in females than in males.

The study identified a significant relationship between the kinetic energy of a fall and fractures of the pelvis (MWU, *p* = 0.0001) and its specific regions, including the pubic bone (MWU, *p* = 0.001), bilateral pubic fractures (MWU, *p* = 0.006), the sacrum (MWU, *p* = 0.001), its middle part (MWU, *p* = 0.034), and the apex (MWU, *p* = 0.03). Additionally, kinetic energy was associated with pubic symphysis rupture (MWU, *p* = 0.018), Bilateral pubic rami fractures (MWU, *p* = 0.042), and upward displacement of pelvic structures (MWU, *p* = 0.015). Higher kinetic energy was observed in groups with these injuries. Every additional meter in height of the fall increased the chances of pelvic fractures by 17.9% (OR = 1.18; 95% CI: 1.076–1.29; LRT: *p* = 0.000011). Furthermore, individuals with shorter stature experienced a higher frequency of pelvic (T-test, *p* = 0.003), pubic bone (T-test, *p* = 0.003), inferior ramus (T-test, *p* = 0.003), superior ramus (T-test, *p* = 0.001), and sacral fractures (T-test, *p* = 0.003).

Accident vs. Suicide

**Figure 4:**
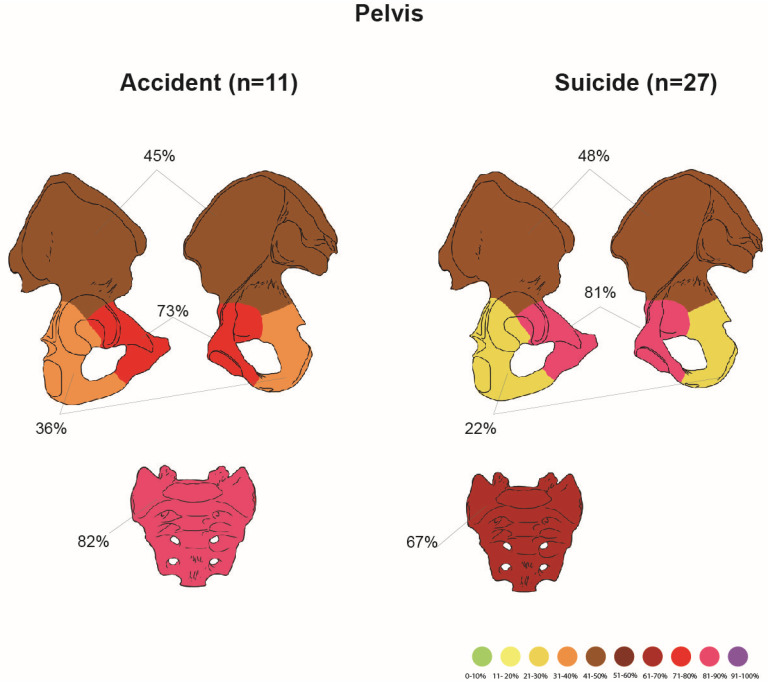
Comparison between accidental and suicidal cases with damage to the pelvis.

Comparison between pelvic injury in accidents and suicides is shown in Figure 4.

There were 11 cases of individuals who had a pelvic fracture as a result of an accident-related fall from a height. Fractures are presented in Table 14.

The cohort of suicides with pelvic injuries included 27 individuals. Fracture frequencies in the suicide group are presented in Table 15.

Cases of suicide, compared to accidents, showed a higher frequency of fractures in the pelvis (χ^2^ test, *p* = 0.004) and pubic bone (χ^2^ test, *p* = 0.005).

Upper Limb

The group of people who suffered a fracture of the upper limb in an accident from height numbered 54. Fracture frequencies are presented in Table 16.

A positive statistical correlation was found between upper limb fractures (MWU, *p* = 0.001), humerus fractures (MWU, *p* = 0.013), and forearm bone fractures (MWU, *p* = 0.038) with the height of the fall. Additionally, other components of the upper limb—scapula (unilateral MWU, *p* = 0.001; bilateral MWU, *p* = 0.004), clavicle (unilateral MWU, *p* = 0.008; bilateral MWU, *p* = 0.003), and ulna (MWU, *p* = 0.044)—showed similar relationships. For every additional meter fallen, the risk of upper limb damage increased 26% (OR = 1.26; 95% CI: 1.062–1.50; LRT: *p* = 0.00036). Meanwhile, no statistically significant correlation was observed between radial bone fractures and the fall category. Furthermore, differences in height categories between groups were more evident for bilateral fractures of the scapula and clavicle.

Statistical analysis indicated that victims with a fractured forearm (either the ulna, radius, or both) (MWU, *p* = 0.002), as well as those with fractures of the ulna (MWU, *p* = 0.01) and radius (MWU, *p* = 0.003), alone, had a lower BMI compared to those without such injuries. Moreover, the kinetic energy from falls was correlated with fractures of the scapula (MWU, *p* = 0.005), bilateral scapula fractures (MWU, *p* = 0.012), clavicle fractures (MWU, *p* = 0.018), bilateral clavicle fractures (MWU, *p* = 0.01), and humerus fractures (MWU, *p* = 0.016).

Accident vs. Suicide

**Figure 5:**
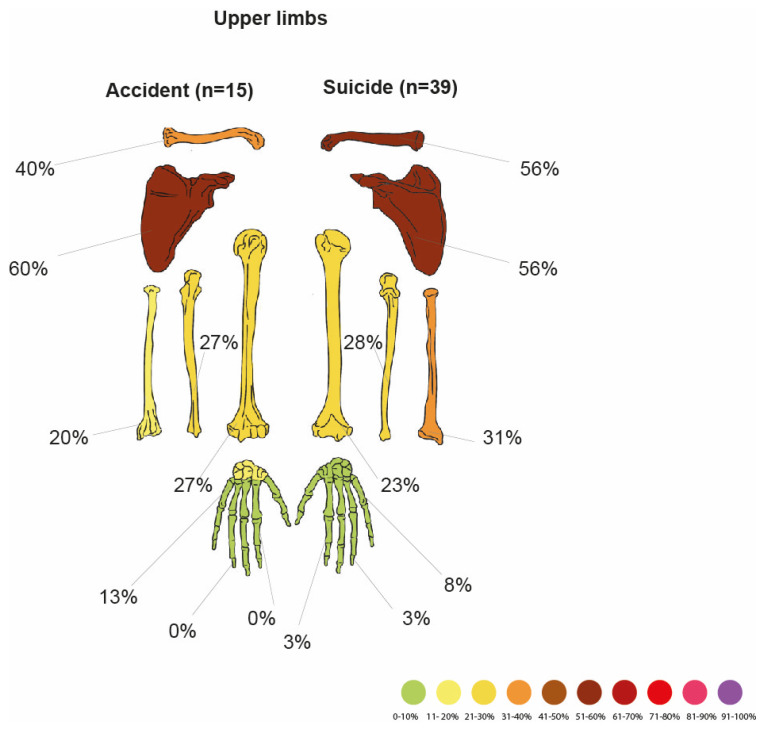
Comparison of upper limb fracture frequencies in accidents and suicides.

Comparison between upper limb injuries in accidents and suicides is shown in Figure 5, Table 17 and Table 18. 

In comparison of these two groups, it was observed that fractures of the upper limb (χ^2^ test, *p* = 0.001), scapula (χ^2^ test, *p* = 0.018), clavicle (χ^2^ test, *p* = 0.004), and forearm (χ^2^ test, *p* = 0.033) are more common in suicides than in accidents. Analysis of logistic regression showed that suicides have an eight times higher risk of upper limb fracture than accidents (OR = 8.089; 95% CI: 2.17–30.19; LRT: *p*= 0.00083).

Lower Limbs

Among 79 victims, fractures in the lower limb (L.L.) occurred in 35 cases (44.30% of all cases). Fracture frequencies are presented in Table 19.

Lower limb fractures were positively correlated with the kinetic energy of the fall (MWU, *p* = 0.000021) and female sex (χ^2^ test, *p* = 0.00158). Fractures of the right (χ^2^ test, *p* = 0.00352) and left (χ^2^ test, *p* = 0.048) femurs, as well as the right (χ^2^ test,, *p* = 0.00148) and left (χ^2^ test, *p* = 0.00025) tibias, right (χ^2^ test, *p* = 0.00148) and left (χ^2^ test, *p* = 0.01631) fibulae were positively associated with the height of the fall. For every meter, the probability of lower limb damage increased 12% (OR = 1.12; 95% CI: 1.022–1.23; LRT: *p* = 0.00086)

The sums of all left (ρ, *p* = 0.000001) and right (ρ, *p* = 0.000016) L.L. fractures were correlated with the height of the fall. Additionally, bilateral lower limb fractures occurred in 18 cases, which were strongly associated with the height of the fall (ANOVA, *p* = 0.0003). Fractures in the right foot (tarsals, metatarsals, and digits) also showed a positive correlation with the height of the fall (ρ, *p* = 0.004278) and, additionally, with a suicidal manner of death (MWU, *p* = 0.0154). We found a statistically significant correlation between left L.L. injury and mental illness (MWU, *p* = 0.00415).

Logistic regression analysis showed that the presence of lower limb fractures increased the probability of pelvic fractures by over three times (OR = 3.21; 95% CI: 1.044–9.86; LRT: *p* = 0.040)—moreover, the best predictors for the L.L damage were upper limb fractures (OR = 14.0; 95% CI: 2.88–68.058; LRT: *p* = 0.000073).

Accident vs. Suicide

**Figure 6:**
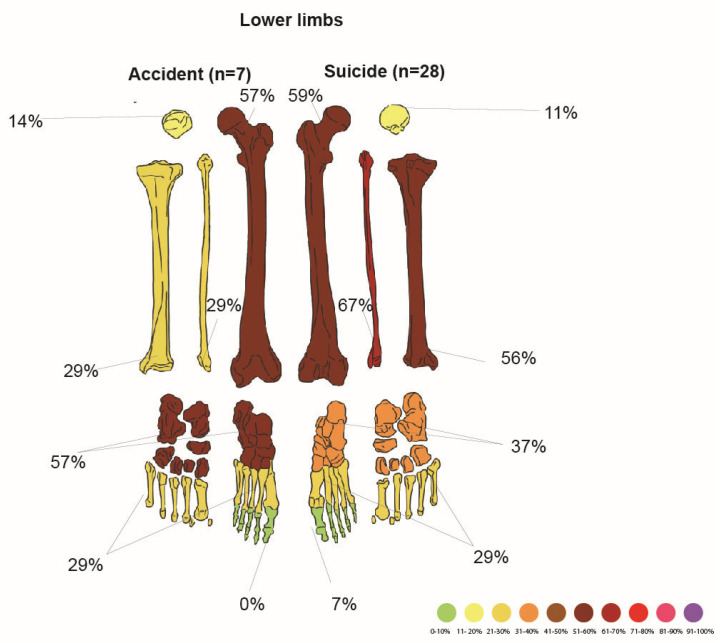
Lower limb fractures in accidents and suicides.

Comparison of lower limb damage in accidental and suicidal fatal falls is shown in Figure 6. Note that the accident group is significantly smaller.

Of the 35 cases with lower limb injuries, 7 were victims of accidents (20% had damaged lower limbs). Fracture frequencies are presented in Table 20.

Of the 35 cases with lower limb injuries, 28 of them occurred as a result of a suicidal fall from a height (80% damaged L.L.). Fractures in the suicides are presented in Table 21.

Injuries to both the right (MWU, *p* = 0.00283) and left (MWU, *p* = 0.000146) L.L. were significantly more common in suicides; this was also true for the right femur (χ^2^ test, *p* = 0.02904). Fractures of both the right (χ^2^ test, *p* = 0.01567) and left (χ^2^ test, *p* = 0.00535) tibia, as well as the right (χ^2^ test, *p* = 0.01567) and left fibula (χ^2^ test, *p* = 0.00078), showed a positive correlation with suicide cases. Bilateral lower limb fractures were found in 16 cases; they were more frequent in suicides than accidents (χ^2^ test, *p* = 0.00278). Logistic regression analysis shows that suicides had a four times higher risk of lower limb fracture than accidents (OR = 3.96; 95% CI: 1.20–13.13; LRT: *p* = 0.022).

## 4. Discussion

Our study population was consistent with previous reports. The majority of participants were male, with a male-to-female ratio of 2.6:1. Men outnumbered women across all categories of fatality, a trend consistent with most prior studies, further reinforcing that FFF primarily affects men. High-risk occupations, particularly in construction, are predominantly held by males. In the case of suicides, men tend to prefer more lethal and violent methods, such as FFF. Contrary to the previous literature, suicides were more prominent in our sample than accidents [15,28,29,30].

The BMI distribution was approximately normal, with most individuals classified as normal weight or overweight and a small proportion as obese or underweight [6].

Alcohol is the most frequently encountered toxin in accidental FFF, as well as a fairly common drug among suicide victims [31]. Our study seems to confirm this notion, with almost ½ of the included accidental and ¼ of the suicidal falls being under the influence. Moreover, statistical analysis revealed that alcohol intoxication was correlated positively with accidents and had no connection with suicides (χ^2^ test, *p* = 0.0133). Alcohol causes postural instability, which increases the risk of accidental FFF [32]. Suicide victims often plan their actions and approach the jump cool-minded. Furthermore, this notion ties in with the observed interplay between alcohol and mental illness. In our study, the mentally ill victims were less likely to test positive for alcohol and more likely to commit suicide. The widespread accessibility of alcohol may also contribute to accidental fatalities.

Previous research has established a correlation between the use of psychoactive substances and upper limb fractures, indicating that uncontrolled flailing movements during falls may contribute to such injuries [11]. Nevertheless, our study did not reproduce this finding. A plausible explanation is the predominance of alcohol consumption within our cohort, as opposed to substances with more pronounced psychostimulant effects. Alcohol, acting as a central nervous system depressant, impairs reaction time and reduces reflex responses [33]. Consequently, individuals under the influence may be less likely to demonstrate protective reflexes during a fall, thereby decreasing the risk of upper limb fractures.

Mental illness typically concerns suicide victims of FFF, with little to no cases of accidents. It is also more common among women. Despite that, FFF are more common among males, both suicidal and accidental [30,34,35]. Our study confirms that over 50% of females have a known psychiatric history, and males outnumber females in both manner-of-death groups. Mental illness was also connected with suicidal intention and the lower height of the fall. Many psychiatric diseases cause suicidal thoughts. However, the exact causes of why one would choose falling over other methods of suicide are a complicated issue [32].

The average fall height of 22.30 m, with a median of 7 m, illustrates the diversity of fall heights among the included victims. Our study seems to be in accordance with the literature, where most fatal falls occur at heights of less than 10 m [7,18]. Similarly, the notion that higher falls are more common among suicide victims was confirmed by our work. Individuals seeking a place to jump from usually choose higher altitudes to ensure lethality [36]. Women had statistically higher falls, most probably because most of them were in the suicide group.

The general injury pattern in our work is broadly consistent with that observed in the literature. It primarily concerns the axial skeleton, with the chest being the most commonly damaged part [37]. Amid many contradictory results, most works agree that rib fractures are the most frequently encountered in FFF. Ribs are directly or indirectly affected in every impact position and type of landing surface. Because of their location and susceptibility, FFF without them is unlikely [9,12,38]. The probability of a rib fracture increases with the height of the fall and kinetic energy, an effect confirmed in our work [39]. This also explains the significantly higher frequency of rib cage fractures among males, as FFF in this group were characterized by greater BMIs. Furthermore, bilateral rib fractures were shown to be more common in suicides and unilateral fractures in accidents. This effect was previously observed and is likely a byproduct of the impact position. Individuals attempting suicide by jumping from a height may employ various techniques, such as running and jumping, stepping off the edge, hanging and releasing, diving head-first, or falling backward intentionally. These controlled or semi-controlled actions often result in a feet-first or buttocks-first landing. In contrast, accidental falls—typically resulting from slips, trips, or unintended loss of balance—more frequently lead to lateral or whole-body impacts, reflecting the unpredictable and unprepared nature of such events. Thus, injuries in suicidal FFF are more symmetrical, and in accidental FFF, they are shifted to one side [38]. The statistical correlation between an increase in rib fractures and mental illness was not described in previous literature. It might stem from differences in impact positions and higher overall altitudes in this group. This effect warrants further investigation.

Skull fractures in the FFF literature have three different distributions depending on the height of the fall, bimodal [5,16], linear rising [6,40], and linear falling [41]. Bimodal distribution—cranial fractures are more prevalent in falls below 10–15 m, less common between 10 and 20 m, and reemerge in falls exceeding 20 m. Experimental and modeling investigations have demonstrated that the initial position predominantly influences the ultimate impact orientation, with impacts to the head being more likely in short falls and landings to the feet or sides more common at intermediate heights due to body angular rotation. At significantly high falls, even impacts to the feet can transmit sufficient energy to induce cranial fractures.

This bimodal pattern contrasts with findings from other studies, which report a linear increase in skull fractures correlating with fall height. Such discrepancies may be attributable to differing ratios of accidental to suicidal falls. Suicides originating from various heights and positions tend to produce more linear distributions, whereas accidental falls—generally lower and more uniform in trajectory—tend to display bimodality. Further comparative research is warranted to substantiate this observation. Finally, some studies report only a decrease in skull fractures with increasing fall height. These might reflect heterogeneity across studies.

Our study falls into the first category, arguing that at lower heights, fallers have little time for the body to rotate. Consequently, the head or upper part of the chest strikes the ground first, leading to cranial fractures. At mid-range (10–20 m), the body has more time to rotate, often landing in a way that spares the skull. However, the potential energy transmitted through the axial skeleton at greater heights becomes significant enough to cause cranial fractures, regardless of the exact impact position.

Skull base fractures typically occur at a distance from the initial point of impact, whereas skull vault fractures are generally found closer to the site of contact. This spatial relationship is influenced by factors such as the victim’s body height and the specific mechanics of the fall. However, the diagnostic value of this distinction is limited. The most common fracture type—linear fractures—can occur both proximally and distally from the impact zone. In such cases, identifying skull base and vault fractures may offer the only anatomical clues to the location and dynamics of the impact [42,43]. The absence of sphenoid fractures among the aforementioned statistical connections may be due to protection from direct impact by the zygomatic arch.

The statistical association between higher BMIs and posterior occipital fractures is quite puzzling, as no such association has been previously described in FFF. Being overweight was even shown to decrease the probability of head and neck injuries [6]. We hypothesize that this effect is best explained by higher potential energies, as per the formula Epot = mgh. Further research is needed to elaborate on those findings.

The inverse association between pelvic and cranial fractures likely reflects biomechanical differences in impact dynamics. During head-first impacts, the cranial region absorbs the majority of kinetic energy, thereby reducing the transmission of force to the pelvis. The facial skeleton received limited attention in previous FFF studies. Some articles have noticed a correlation between the height of the fall and the resulting fractures [14,38]. But this was not universally attested [44]. Our study found statistical correlations between maxillary and mandible fractures, the height of the fall, and kinetic energy, suggesting that facial skeleton injuries may provide helpful forensic clues. Impacts other than feet, knees, or buttocks first should, at least in some cases, injure salient elements of the facial skeleton [45]. This relationship also suggests that the facial skeleton was not damaged in the “second impact” after bouncing off the ground.

The spine was the second most frequently injured region in our study, a finding that differs from most reports in the current literature [46,47]. This discrepancy may be attributed to the use of postmortem PMCT, which enhances the detection of deep fractures.

Spinal fractures are known to correlate with fall height, as fall-related injuries predominantly affect the axial skeleton [44,48]. Given this, we expected kinetic energy to be associated with spinal injuries. Interestingly, when analyzing specific spinal regions, only the lumbar vertebrae showed a significant correlation with fall height. This stands with previous studies on living individuals that have identified the thoracolumbar junction as the most commonly fractured area in fall-related injuries [49]. No correlations were found for specific parts of each vertebra, suggesting that their relation with other variables is either complex or unpredictable.

The strong association between spinal and thoracic trauma revealed by logistic regression is anatomically and biomechanically plausible, as the axial skeleton is primarily affected in falls from height. In all impact orientations, the vertebrae absorb a substantial portion of the impact energy. Pelvic fractures pose a significant risk due to the potential for severe hemorrhage. They typically occur in feet-first, buttocks-first, or whole-body impacts, which arise from either direct horizontal deceleration or indirect vertical force transmission, primarily through the femur. Consequently, these injuries are often centered around major joints— sacroiliac joints and pubic symphysis, although they differ structurally, being partly fibrous and cartilaginous, respectively [9,29,38]. Pelvis fractures increase with the height of the fall and are more common among suicides [50]. Although not all studies find this relationship [18].

Our study confirmed that the incidence of pelvic (*p* = 0.001), pubic (*p* = 0.003), and sacral (*p* = 0.008) fractures increases with fall height. However, kinetic energy showed a stronger correlation, as expected given its critical role in trauma severity. Nearly all severe pelvic injuries were linked to higher kinetic energy levels. Lower body height is connected to lower bone mineral density [51]. This might explain why shorter individuals are statistically more likely to experience pelvic fractures.

Logistic regression identified key predictors of skeletal injuries in fatal falls. Both body mass and fall height significantly influenced pelvic fractures; each extra kilogram decreased pelvic injury odds by 5.4% (OR = 0.95; *p* = 0.0019), while each additional meter increased the odds by 17.9% (OR = 1.18; *p* < 0.001). The presence of lower limb fractures more than tripled the odds of pelvic fractures (OR = 3.21; *p* = 0.040). Fall height also predicted lower limb injuries (OR = 1.12/meter; *p* < 0.001), with upper limb fractures strongly linked (OR = 14.0; *p* < 0.001). Suicides significantly raised the odds of upper limb (OR = 8.09; *p* < 0.001) and lower limb fractures (OR = 3.96; *p* = 0.022) compared to accidental falls. Thoracic trauma markedly increased the likelihood of spinal injury, with vertebral fractures nearly tenfold when sternocostal fractures were present (OR = 9.67; *p* = 0.0024).

Interestingly, our findings contradict previous research suggesting that obesity serves as a protective factor against specific injuries, including pelvic fractures [52]. This discrepancy may be explained by the heterogeneity of the studied populations and the role of body fat as padding. Additionally, our study reinforces the well-documented association between pelvic fractures and suicidal falls. Possibly because of the association between higher falls and suicides explored earlier.

Bilateral pubic rami fracture was present in ⅕ of all pelvic injuries in our study group, which is much more common than in clinical literature. As fatal falls tend to happen with greater kinetic energy and more extensive injuries, this is not surprising [53]. The low incidence of fallers’ fractures aligns with the existing literature, establishing it as a rare but characteristic finding [23]. No statistical correlations were found for these injuries.

According to the Young and Burgess classification, lateral compression injuries were the most prevalent in our study, consistent with the current literature. In real-life falls, it is rare for individuals to land in a perfectly vertical or horizontal posture with symmetrical force distribution. Instead, victims often strike the ground on their side or buttock, introducing a lateral force vector that drives inward rotation of the hemipelvis, leading to characteristic coronal-plane fractures of the pubic rami and sacrum.

Anterior–posterior compression injuries were the second most frequent. This is likely due to the wide variability in landing positions during high-energy falls. Victims who land in a semi-upright, supine, or extended-leg position may transmit front-to-back forces through the pelvis, resulting in symphysis pubis widening and disruption of the pelvic floor and sacroiliac ligaments, consistent with APC injury patterns. Such mechanisms are commonly observed in falls from height involving axial loading combined with torso hyperextension or leg abduction.

Vertical shear injuries, caused by asymmetrical axial loading (e.g., landing on one leg), were rare. This supports the notion that such unilateral, high-magnitude impacts are relatively uncommon in fatal falls. Likewise, posterior–anterior compression patterns—resulting from a posteriorly directed force—are infrequent.

Upper limb injuries remain highly contested in the literature. Some studies see them as almost random and without a specific pattern [39,41], and others connect them with the height of the fall [12,15,54]. Our study aligns with the latter. The body’s defense mechanisms include extending the arms in preparation for impact, which increases the risk of fractures in both the first and second impacts. Although a feet-first landing is the most common, any other position would also involve this reflex [55]. The strong correlation between bilateral clavicle and scapular fractures, fall height, and kinetic energy may be attributed to their proximity to the axial skeleton and the body’s impact position. This suggests that these fractures could serve as potential markers for estimating fall altitude. Additionally, given that suicidal falls typically involve higher kinetic energies, this factor likely contributes to the increased frequency of clavicle and scapular fractures observed in suicides. Suicides had an increased probability of upper limb fractures compared to accidents (OR = 8.089). Additionally, upper limb fractures were the best predictor for lower limb fractures. These relations might stem from the overall higher kinetic energy of suicide falls.

In the study, fractures of the pelvis and its bones, excluding the iliac bone, were more frequently noted in females than in males. Fractures of the forearm and radial bone showed analogous patterns. Furthermore, we observed a correlation between lower BMIs and pelvic injuries. These relationships can be explained by the higher risk of osteoporosis in females and the typical fracture sites, namely the pelvis and forearm. Moreover, lower BMI may reflect frail bones [56].

As expected, fractures of the lower limbs—both unilateral and bilateral—were primarily influenced by the height of the fall and the associated kinetic energy. Interestingly, the presence of such fractures was the most distinguishing factor between low and very high falls, suggesting that lower limb fractures could serve as a valuable indicator in altitude estimation.

Notably, in suicides, both individual and bilateral lower limb fractures were significantly more frequent than in accidental falls (OR = 3.96), likely due to differences in impact positioning. Suicide victims may initiate a free fall through multiple mechanisms, including running and jumping, stepping over an edge, hanging and releasing, or leaning forward from a height. The majority of these actions lead to a descent feet-first, which, in the absence of substantial body rotation, probably explains the high incidence of lower limb injuries documented in such cases. This aligns with previous literature. Some studies have proposed that fractures of the dominant lower limb are more common in fatal fall suicides, as the final step before jumping is typically taken with the dominant leg, increasing its likelihood of striking the ground first [57,58]. Our findings reinforce this idea, showing that nearly all lower limb bones exhibited a correlation with fall height and suicidal intent, except for the left femur and foot. Specifically, fractures of the right femur and foot were more prevalent in suicides but not in accidents, possibly reflecting the dominance-related mechanism mentioned earlier. However, recent studies have not consistently supported this association [12], highlighting the need for further research to clarify these findings.

The discrepancies between our results and earlier studies may arise from methodological and population differences. In contrast to many previous reports that included mixed fall types or impacts on deformable surfaces, our dataset was restricted to free falls onto rigid, non-deformable substrates, which likely resulted in more extensive skeletal trauma. Moreover, the use of high-resolution PMCT enabled the identification of subtle or incomplete fractures that might have been missed in autopsy-based investigations. Differences in demographic structure—particularly age distribution and the predominance of urban falls in our material—may also have influenced injury configurations. Together, these factors could explain part of the variation in reported fracture prevalence and distribution across studies.

Our study had several limitations. The sample size was relatively small, and many cases were excluded due to missing data, such as fall height or an unclear cause of death. This may have introduced bias in the reported fracture frequencies and their statistical correlations. Rare fractures might have been overlooked. Additionally, PMCT has limitations in detecting fractures in thin or less ossified bones, including the ethmoid bone. Given the unique nature of forensic cases, studied populations tend to be heterogeneous, making it challenging to generalize findings across different groups. This study standardized the surface type to impact on hard, non-deformable surfaces. Fracture patterns resulting from deformable or yielding surfaces require separate examination to determine potential differences. Toxicological parameters, particularly alcohol levels, were analyzed in relation to the manner of death. The body position at impact could not be reliably documented retrospectively, as all included fatal falls lacked direct witnesses; therefore, it was not assessed. This limitation may have influenced the interpretation of specific fracture patterns observed in our study.

Furthermore, free-fall trauma remains unpredictable due to the multitude of variables influencing each incident. Statistical analysis may identify certain fractures and correlations as more characteristic of suicidal or accidental falls; however, the overall skeletal injury patterns exhibit considerable overlap between the two groups. Consequently, the correlations established in this study should not be regarded as universally diagnostic. Instead, our findings should be interpreted as offering a set of supportive indicators—arguments for or against a particular manner of death—within the broader context of forensic evaluation. This study primarily analyzed fracture frequencies in fatal falls for each bone. Additionally, we identified novel relationships between key variables and specific injuries, including correlations between facial skeletal fractures and fall height, posterior occipital bone fractures and BMI, and the association between the cause of the fall and the fractured side of the body. Many of our findings align with previous research, and in several cases, we were able to refine them to the level of individual bones. Furthermore, we uncovered new insights that may help differentiate between suicides and accidental falls.

From a clinical perspective, our findings might be helpful for emergency care and trauma support. The most essential findings are presented in Table 22.

Although the finding that the number and severity of injuries increase with both the height of the fall and the resulting increase in kinetic energy is not novel, our study identified several anatomical regions that are particularly prone to injury. These include the facial skeleton, the occipital and temporal bones, the lumbar vertebrae, the ribs, and the lower limb bones. Recognizing these vulnerable areas may help clinicians assess and plan treatment for victims of free-fall injuries.

Understanding how intrinsic factors such as BMI and sex influence injury patterns can further inform risk assessment and clinical expectations. Emergency physicians and trauma care personnel should also be aware of the distinct differences between suicidal and accidental fall injuries. Suicidal falls tend to produce more severe, bilateral, and complex fractures; therefore, patients presenting after a suspected suicide attempt by fall should be managed as high-priority trauma cases. Extensive injuries involving both the axial and appendicular skeletons are to be anticipated. Notably, facial fractures may compromise airway patency, while spinal trauma often requires urgent stabilization.

In subsequent care, the evaluation of injury distribution may also contribute to the differentiation between suicidal and accidental mechanisms. Because suicidal individuals may sometimes misrepresent their intent, awareness of these characteristic patterns may aid both in accurate medico-legal interpretation and in the planning of appropriate psychiatric follow-up and preventive interventions. Postmortem CT is not the sole emerging technique capable of augmenting fall investigations. Biomechanical experiments have been successfully employed to distinguish between conflicting reports of the incident (e.g., accidental versus homicidal falls). Furthermore, computer-based simulations have recreated fracture patterns that were subsequently verified during autopsy in numerous cases. Similar results were achieved by Henningsen et al.: subject-specific finite-element head models were used to predict PMCT and autopsy findings, with relative success. Collectively, these methodologies underscore promising new avenues for reconstructing and interpreting free-fall events [59,60,61].

Future studies should further explore bone fractures using post-mortem computed tomography. This approach will enable forensic experts to replace ambiguous terms, such as “limb fracture,” with more precise descriptions. Recent advancements in artificial intelligence are promising in this regard, with AI models being used to assess fracture morphology [62].

Additionally, the correlation between body mass index and fractures of the skull and pelvis needs further investigation. Our findings related to the maxilla and mandible should also be examined more closely. The effects of intoxication and mental illness on fracture patterns should be examined in greater detail. Ongoing research should aim to identify new markers for accidental and suicidal fatal falls. The topic of homicidal fatal falls requires comprehensive study, as such cases are rare; conducting a multicenter study could provide a larger sample size for analysis.

## 5. Conclusions

FFFs are commonly encountered phenomena in forensic practice. Many variables, such as the victim’s sex, BMI, intoxication, height of the fall, and mental illness, influence injury patterns. Injuries in free fatal falls tend to focus on the axial skeleton. Suicides experience more severe, bilateral fractures, often involving the pelvis and limbs, while accidents tend to have unilateral injuries with rare limb involvement. Fracture frequencies rise with height and the kinetic energy of the fall.

This study demonstrated that victims of fatal free falls present distinct skeletal injury patterns, which differ between suicides and accidents. Postmortem computed tomography proved to be a highly valuable complement to conventional autopsy, enabling the detection of complex fractures that may otherwise remain underreported, particularly in the skull base and pelvis. From a clinical perspective, the identification of specific trauma configurations may improve diagnostic precision in emergency medicine and trauma care, supporting the differentiation of injury mechanisms in survivors of high-energy falls. In forensic practice, the combined use of autopsy and PMCT enhances the reliability of medico-legal evaluations, allowing for more accurate determination of the manner of death and contributing to the reconstruction of circumstances in disputed cases. Overall, integrating PMCT into routine forensic protocols should be considered a standard approach to increase diagnostic accuracy and medico-legal certainty.

## Figures and Tables

**Table 1:** General Information about the Study Population. Note a high percentage of mental illnesses and alcohol intoxication cases.

Data	All Cases	% of Cases	Female	% Among Women	Male	% Among Men
Cases	79	100	22	100	57	100
Accident	36	45.57	4	18.18	32	56.14
Suicide	43	54.43	18	81.82	25	43.86
Height of the fall						
≤3 m	20	25.32	2	9.09	18	31.58
3 < x < 6 m	8	10.13	2	9.09	6	10.53
6 ≤ x < 10 m	21	26.58	7	31.82	14	24.56
10 m ≤ x < 15 m	14	17.72	4	18.18	10	17.54
15 m ≤ x < 20 m	3	3.80	2	9.09	1	1.75
20 m ≤ x < 25 m	5	6.33	2	9.09	3	5.26
25 m ≤ x < 30 m	4	5.06	2	9.09	2	3.51
30 m ≤ x	4	5.06	1	4.55	3	5.26
Time of death from the fall						
Died on site	46	58.23	13	59.09	33	57.89
<1 h	6	7.59	3	13.64	3	5.26
Several hours	13	16.46	1	4.55	12	21.05
1–2 days	3	3.80	2	9.09	1	1.75
3–4 days	5	6.33	1	4.55	4	7.02
5–10 days	3	3.80	1	4.55	2	3.51
>10 days	2	2.53	0	0.00	2	3.51
Unmarked	1	1.27	1	4.55	0	0.00
Intrinsic Variables						
Alcohol present	26	32.91	4	18.18	22	38.60
Accident	17	21.52	1	4.55	16	28.07
Suicide	9	11.39	3	13.64	6	10.53
Documented mental illness	20	25.32	11	50.00	9	15.79
Alcoholism	4	5.06	0	0.00	4	7.02
Dementia	2	2.53	2	9.09	0	0.00
Depression	5	6.33	3	13.64	2	3.51
Schizophrenia	4	5.06	1	4.55	3	5.26
Unspecified	5	6.33	5	22.73	0	0.00
No mental illness	59	74.68	11	50.00	48	84.21

**Table 2:** Accidental deaths across main variables. Note the higher concentration of accidental deaths at lower altitudes and prominent alcohol intoxication cases.

Data	All Cases	% of Cases	Female	% Among Women	Male	% Among Men
Accident	36	100	4	100	32	100
Height of the fall						
≤3 m	15	41.67	0	0.00	15	46.88
3 < x < 6 m	6	16.67	2	50.00	4	12.50
6 ≤ x < 10 m	6	16.67	0	0.00	6	18.75
10 m ≤ x < 15 m	7	19.44	2	50.00	5	15.63
15 m ≤ x < 20 m	0	0.00	0	0.00	0	0.00
20 m ≤ x < 25 m	1	2.78	0	0.00	1	3.13
25 m ≤ x < 30 m	0	0.00	0	0.00	0	0.00
30 m ≤ x	1	2.78	0	0.00	1	3.13
Intrinsic Variables						
Alcohol present	17	47.22	1	25.00	16	50.00
Documented mental illness	3	8.33	1	25.00	2	6.25
Alcoholism	2	5.56	0	0.00	2	6.25
Dementia	1	2.78	1	25.00	0	0.00
Depression	0	0.00	0	0.00	0	0.00
Schizophrenia	0	0.00	0	0.00	0	0.00
Unmarked	0	0.00	0	0.00	0	0.00
No mental illness	33	91.67	3	75.00	30	93.75

**Table 3:** Suicides across main variables. Note that suicides have a higher rate of psychiatric diseases as well as higher altitudes.

Data	All Cases	% of Cases	Female	% Among Women	Male	% Among Men
Suicide	43	100	18	100	25	100
Height of the fall						
≤3 m	5	11.63	2	11.11	3	12.00
3 < x < 6 m	2	4.65	0	0.00	2	8.00
6 ≤ x < 10 m	15	34.88	7	38.89	8	32.00
10 m ≤ x < 15 m	7	16.28	2	11.11	5	20.00
15 m ≤ x < 20 m	3	6.98	2	11.11	1	4.00
20 m ≤ x < 25 m	4	9.30	2	11.11	2	8.00
25 m ≤ x < 30 m	4	9.30	2	11.11	2	8.00
30 m ≤ x	3	6.98	1	5.56	2	8.00
Intrinsic Variables						0.00
Alcohol present	9	20.93	3	16.67	6	24.00
Documented mental illness	17	39.53	10	55.56	7	28.00
Alcoholism	2	4.65	0	0.00	2	8.00
Dementia	1	2.33	1	5.56	0	0.00
Depression	5	11.63	3	16.67	2	8.00
Schizophrenia	4	9.30	1	5.56	3	12.00
Unmarked	5	11.63	5	27.78	0	0.00
No mental illness	26	60.47	8	44.44	18	72.00

**Table 4:** Skull fracture frequencies in the studied population.

Damaged Structure	All Cases	% of Cases	Bilateral/Complex Injury
Sphenoid bone	34	72.34	-
Body of the sphenoid bone	17	36.17	-
Greater wing of the sphenoid bone	26	55.31	9
Pterygoid process of the sphenoid bone	2	4.25	2
Temporal bone	30	63.82	12
Petrous part of sphenoid bone	20	42.55	10
Squamous part of sphenoid bone	25	53.19	5
Occipital bone	27	57.44	-
Posterior part of occipital bone	15	31.91	8—Anterior + posterior part
Anterior part of occipital bone	20	42.55	
Maxilla	19	40.42	8
Frontal bone	16	34.04	-
Parietal bone	20	42.55	7
Nasal bone	8	17.02	6
Ethmoid bone	8	17.02	-
Palatine bone	4	8.51	2
Vomer	4	8.51	-
Zygomatic bone	12	25.53	3
Separation of the zygomatic bone	5	10.63	2
Zygomatic arch	19	40.42	3
Mandible	14	29.78	
Body of the mandible	12	25.53	1—Body + Left and Right ramus
Ramus of the mandible	5	10.63	

**Table 7:** Rib cage injuries in the studied population.

Damaged Structure	All of the Cases	% of Cases	The Most Common Type of Injury
Rib cage	70	100	-
Sternum	40	57.14	12.5% − manubrium + sternum
manubrium	26	37.14	80.77% of fractures − single fracture
body	19	27.14	78.95% of fractures − single fracture
Rib	68	97.14	76.47% of fractures − bilateral
Right ribs	58	82.86	70.18% of fractures − single fracture
Left ribs	62	88.57	69.93% of fractures − single fracture

**Table 8:** Rib cage fracture frequencies in the accident group.

Damaged Structure	All of the Cases	% of Cases	The Most Common Type of Injury
Rib cage	27	100	-
Sternum	13	48.15	23.08% − manubrium + sternum
manubrium	7	25.93	85.71% of fractures − single fracture
body	9	33.33	88.89% of fractures − single fracture
Rib	25	92.59	60.00% of fractures − bilateral
Right ribs	18	66.67	66.66% of fractures − single fracture
Left ribs	22	81.48	70.23% of fractures − single fracture

**Table 9:** Rib cage fracture frequencies in the suicide group.

Damaged Structure	All of the Cases	% of Cases	The Most Common Type of Injury
Rib cage	43	100	-
Sternum	27	62.79	7.41% − manubrium + sternum
manubrium	19	44.19	78.95% of fractures − single fracture
body	10	23.26	70.00% of fractures − single fracture
Rib	43	100	83.72% of fractures − bilateral
Right ribs	39	90.7	71.43% of fractures − single fracture
Left ribs	40	93.02	69.80% of fractures − single fracture

**Table 10:** Spinal injuries in the studied population.

Damaged Structure	All Cases with Vertebral Injury	% of Cases with Vertebral Injury	The Most Injured Vertebra of the Level	The Most Common Type of Injury
Vertebral injury	61	100%	L2—50.82% of cases, L3—50.82% of cases	-
Cervical vertebrae	19	31.15%	C2—47.37% cases	-
C1	4	6.56%	-	-
Anterior arch	1	-	-	-
Posterior arch	2			
Left transverse process	1			
Right transverse process	0			
Bilateral transverse process fracture	0			
C2	9	14.75%	-	-
single fracture of the body	2	-	-	-
Multiple fractures (compression) of the body	0			
Arch	6			
Left transverse process	1			
Right transverse process	0			
Bilateral transverse process fracture	0			
Spinal process	3			
C3–C7	15	24.59%	C7—46.66% of cases	73.08% of fractured vertebras—Spinous process fracture
Thoracic vertebrae	45	73.77%	Th7—44.44% of cases, Th8—44.44% of cases	52.50% of fractured vertebras Th1–Th10—Spinous process fracture, 47.62% of fractured vertebras Th11–Th12—multipe fratures of the body
Lumbar vertebrae	44	72.13%	L2—70.45% of cases, L3—70.45% of cases	85.16% of fractured vertebras—fracture of the costal process
Rupture of the spine	2	6.56%	-	-
Cervical vertebrae	0	0%	-	-
Thoracic vertebrae	2	3.28%	Th7–Th8 and Th8–Th9	
Lumbar vertebrae	2	3.28%	L4–L5 and L1–L2	
Coccyx	3	4.92%	-	-

**Table 11:** Spinal fractures in the accident group.

Damaged Structure	All Cases with Vertebral Injury	% of Cases with Vertebral Injury	The Most Injured Vertebra of the Level	The Most Common Type of Injury
Vertebral injury	26	100%	L1—38.46% of cases, L2—38.46% of cases, L3—38.46% of cases	-
Cervical vertebrae	9	34.62%	C2—44.44% of cases, C7—44.44% of cases	-
C1	1	3.85%	-	-
Anterior arch	0	-	-	-
Posterior arch	1			
Left transverse process	0			
Right transverse process	0			
Bilateral transverse process fracture	0			
C2	4	15.38%	-	-
single fracture of the body	1	-	-	-
Multiple fractures (compression) of the body	0			
Arch	3			
Left transverse process	0			
Right transverse process	0			
Bilateral transverse process fracture	0			
Spinal process	1			
C3–C7	5	19.23%	C7—80.00% of cases	69.23% of fractured vertebras—spinous process fracture
Thoracic vertebrae	19	73.08%	Th7—42.11% of cases	41.51% of fractured vertebras Th1–Th10—Spinous process fracture, 88.89% of fractured vertebras Th11–Th12—fratures of the body
Lumbar vertebrae	15	57.69%	L1—66.67% of cases, L2—66.67% of cases, L3—66.67% of cases	87.8% of fractured vertebras—fracture of costal process
Rupture of the spine	0	0%	-	-
Cervical vertebrae	0	0%	-	-
Thoracic vertebrae	0	0%	-	
Lumbar vertebrae	0	0%	-	
Coccyx	2	7.69%	-	-

**Table 12:** Spinal injuries in the suicide group.

Damaged Structure	All Cases with Vertebral Injury	% of Cases with Vertebral Injury	The Most Injured Vertebra of the Level	The Most Common Type of Injury
Vertebral injury	35	100%	L2—60.00% of cases, L3—60.00% of cases	-
Cervical vertebrae	10	28.57%	C2—50.00% of cases	-
C1	3	8.57%	-	-
Anterior arch	1	-	-	-
Posterior arch	1			
Left transverse process	1			
Right transverse process	0			
Bilateral transverse process fracture	0			
C2	5	14.28%	-	-
single fracture of the body	1	-	-	-
Multiple fractures (compression) of the body	0			
Arch	3			
Left transverse process	1			
Right transverse process	0			
Bilateral transverse process fracture	0			
Spinal process	2			
C3–C7	8	22.86%	C7—87.5% of cases	76.92% of fractured vertebras—Spinous process fracture
Thoracic vertebrae	26	74.29%	Th8—50.00% of cases, Th10—50.00% of cases	58.49% of fractured vertebras Th1–Th10—Spinous process fracture, 50% of fractured vertebras Th11–Th12—multipe fratures of the body
Lumbar vertebrae	29	82.26%	L2—72.41% of cases, L3—72.41% of cases	83.91% of fractured vertebras—fracture of costal process
Rupture of the spine	2	11.43%	-	-
Cervical vertebrae	0	0%	-	-
Thoracic vertebrae	2	5.71%	Th7–Th8 and Th8–Th9	
Lumbar vertebrae	2	5.71%	L4–L5 and L1–L2	
Coccyx	1	2.86%	-	-

**Table 13:** Pelvic fracture frequencies in the study population.

Damaged Structure	All Cases	% of Cases	Bilateral	The Most Common Type of Injury
Pubis	31	81.58	18	-
Superior pubic ramus	27	71.05	14	73.17% of fractures—comminuted fracture
Inferior pubic ramus	27	71.05	15	76.19% of fractures—comminuted fracture
Body of pubis	2	5.26	-	-
Bilateral pubic rami fracture	8	21.05	-	-
Rupture of symphysis pubis	6	15.79	-	-
Ilium	18	47.37	2	-
ALa of ilium	15	39.47	0	53.33% of fractures—monofragmentary fracture
Body of ilium	10	26.32	2	58.33% of fractures—monofragmentary fracture
Ischium	10	26.32	3	-
Body of ischium	5	13.16	1	83.33% of fractures—monofragmentary fracture
Ischial tuberosity	4	10.53	0	50.00% of fractures—comminuted fracture
Ramus of ischium	4	10.53	2	83.33% of fractures—monofragmentary fracture
Sacrum	27	71.05	-	8—complex injury
Base of sacrum	5	13.16	-	5—comminuted fracture
Central part of sacrum	8	21.05	-	4—vertical fracture
Lateral part of sacrum	24	63.16	8	91.66% of fractures—vertical fracture, 1 fragmentacja
Apex of sacrum	7	18.42	-	85.71% of fractures—comminuted fracture
Sacroiliac joint	10	26.32	3	-
Faller’s fracture	1	2.63	-	-
Upward displacement of part of the pelvis	5	13.16	-	-
Flexion fracture	12	31.58	-	-
Young–Burgess classification	38	100.00		
LC compression	23	60.53	-	-
APC compression	10	26.32	-	-
VS compression	2	5.26	-	-
PAC compression	3	7.89	-	-

**Table 14:** Pelvis fractures in the accident group.

Damaged Structure	All Cases	% of Cases	Bilateral	The Most Common Type of Injury
Pubis	8	72.73	7	-
Superior pubic ramus	7	63.64	6	61.54% of fractures—comminuted fracture
Inferior pubic ramus	6	54.55	6	66.67% of fractures—comminuted fracture
Body of pubis	0	0.00	0	-
Bilateral pubic rami fracture	4	36.36	-	-
Rupture of symphysis pubis	1	9.09	-	-
Ilium	5	45.45	0	-
Ala of ilium	4	36.36	0	75.55% of fractures—comminuted fracture
Body of ilium	3	27.27	0	66.67% of fractures—single fracture
Ischium	4	36.36	0	-
Body of ischium	2	18.18	0	100.00% of fractures—single fracture
Ischial tuberosity	2	18.18	0	100.00% of fractures—single fracture
Ramus of ischium	1	9.09	0	100.00% of fractures—single fracture
Sacrum	9	81.82	-	3—complex injury
Base of sacrum	1	9.09	-	100.00% of fractures—comminuted fracture
Central part of sacrum	2	18.18	-	50.00% of fractures—horizontal fracture
Lateral part of sacrum	8	72.73	3	87.50% of fractures—vertical fracture
Apex of sacrum	3	27.27	-	66.67% of fractures—comminuted fracture
Sacroiliac joint	2	18.18	0	-
Faller’s fracture	1	9.09	-	-
Upward displacement of part of the pelvis	1	9.09	-	-
Flexion fracture	4	36.36	-	-
Young–Burgess classification	11	100.00		
LC compression	7	63.64	-	-
APC compression	2	18.18	-	-
VS compression	0	0.00	-	-
PAC compression	2	18.18	-	-

**Table 15:** Fracture frequencies in the suicide group.

Damaged Structure	All Cases	% of Cases	Bilateral	The Most Common Type of Injury
Pubis	23	85.19	11	-
Superior pubic ramus	20	74.07	8	78.57% of fractures—comminuted fracture
Inferior pubic ramus	19	70.37	9	85.71% of fractures—comminuted fracture
Body of pubis	2	7.41	0	100.00% of fractures—single fracture
Bilateral pubic rami fracture	4	14.81	-	-
Rupture of symphysis pubis	5	18.52	-	-
Ilium	13	48.15	2	-
Ala of ilium	11	40.74	0	81.82% of fractures—singular fracture
Body of ilium	8	29.63	1	55.56% of fractures—singular fracture
Ischium	6	22.22	3	-
Body of ischium	3	11.11	1	75.00% of fractures—singular fracture
Ischial tuberosity	2	7.41	0	100% of fractures—comminuted fracture
Ramus of ischium	5	18.52	2	80.00% of fractures—singular fracture
Sacrum	18	66.67	-	-
Base of sacrum	4	14.81	-	100% of fractures—comminuted fracture
Central part of sacrum	6	22.22	-	66.67% of fractures—comminuted fracture
Lateral part of sacrum	16	59.26	5	93.75% of fractures—vertical fracture
Apex of sacrum	4	14.81	-	100% of fractures—comminuted fracture
Sacroiliac joint	8	29.63	3	-
Faller’s fracture	0	0.00	-	-
Upward displacement of part of the pelvis	4	14.81	-	-
Flexion fracture	8	29.63	-	-
Young–Burgess classification	27	100.00		
LC compression	16	59.26	-	-
APC compression	8	29.63	-	-
VS compression	2	7.41	-	-
PAC compression	1	3.70	-	-

**Table 16:** Upper limb fractures in the studied population.

Damaged Structure	All Cases	Right	Left	% of Cases	Bilateral
Clavicle	28	18 (61.11%—monofragmentary fracture)	16(50.00%—comminuted fracture)	51.85	6
Scapula	31	25 (60.00%—monofragmentary fracture)	15 (60.00%—comminuted fracture)	57.41	9
Humerus	13	6 (33.33%—single fracture of the body)	9 (33.33%—comminuted fracture of proximal end)	24.07	2
Ulna	15	8 (37.50%—comminuted fracture of distal end)	9 (44.44%—comminuted fracture of distal end)	27.78	2
Radius	15	6 (100.00%—comminuted fracture of distal end)	12 (66.66%—comminuted fracture of distal end)	27.78	4
Carpal bones	5	3	2	9.26	0
Metacarpal bones	1	1	0	1.85	0
Phalanges	1	1	0	1.85	0

**Table 17:** Upper limb fractures in the accident group.

Damaged Structure	All Cases	Right	Left	% of Cases	Bilateral
Clavicle	6	2 (50.00%—comminuted fracture)	5 (60.00%—comminuted fracture)		1
Scapula	9	7 (71.43%—monofragmentary fracture)	4 (75.00%—comminuted fracture)		2
Humerus	4	2 (comminuted fracture and single fracture of proximal end)	3 (66.66%—comminuted fracture of proximal end)		1
Ulna	4	2 (single fracture 50.00% of—distal end, 50.00% of proximal end)	3 (66.66%—comminuted fracture of distal end)		1
Radius	3	1 (comminuted fracture of distal end)	2 (50.00%—comminuted fracture of distal end, 50.00%—single fracture of proximal end)		0
Carpal bones	2	0	2		0
Metacarpal bones	0	0	0		0
Phalanges	0	0	0		0

**Table 18:** Upper limb fractures in the suicide group.

Damaged Structure	All Cases	Right	Left	% of Cases	Bilateral
Clavicle	22	16 (62.50%—single fracture)	11 (54.55%—comminuted fracture)		5
Scapula	22	18 (55.56%—single fracture)	11 (54.55%—comminuted fracture)		7
Humerus	9	4 (50.00%—single fracture of the body)	6 (33.33%—comminuted fracture of the body, 33.33%—single fracture of the body, 33.33%—comminuted fracture of the distal end)		1
Ulna	11	6 (50.00%—comminuted fracture of distal end)	6 (33.33%—comminuted fracture of distal end, 33.33%—comminuted fracture of proximal end)		1
Radius	12	6 (83.33%—comminuted fracture of distal end)	10 (70.00%—comminuted fracture of distal end)		0
Carpal bones	3	3	0		0
Metacarpal bones	1	1	0		0
Phalanges	0	0	0		0

**Table 19:** Lower limb fractures in the studied population.

Damaged Structure	All Cases with Lower Limb Injury	Right	Left	% Damaged Lower Limb	Bilateral
Lower limb	35	20	33	100	18
Femur	20	12 (58.33% of fractures—comminuted fracture of distal end)	16 (31.25% of fractures—comminuted fracture of shaft, 31.25% of fractures—single fracture of proximal end)	57.14	8
Patella	4	3 (66.67% of fractures—comminuted fracture)	2 (50.00% of fractures—single fracture, 50.00% of fractures—comminuted fracture)	11.43	1
Tibia	17	10 (40.00% of fractures—comminuted fracture of proximal end)	15 (33.33% of fractures—comminuted fracture of proximal end)	48.57	8
Fibula	20	10 (40.00% of fractures—comminuted fracture of proximal end)	15 (40.00% of fractures—comminuted fracture of proximal end)	57.14	5
Tarsal bones	14	7 (40.00% of fractures—fracture of calcaneus, 40.00% of fractures—fracture of navicular)	12 (50.00% of fractures—fractures of calcaneus)	40	5
Metatarsals	10	6	5	28.57	1
Phalanges	2	2	0	5.71	0

**Table 20:** Lower limb fractures in the accidental fatal falls.

Damaged Structure	All Cases with Lower Limb Injury	Right	Left	% Damaged Lower Limb	Bilateral
Lower limb	7	3	6	100	2
Femur	4	2 (50% of fractures—comminuted fracture of distal end, 50% of fractures—comminuted fracture of shaft)	4 (50% of fractures—fracture of proximal end)	57.14	2
Patella	1	1 (100% of fractures—single fracture)	0	14.29	0
Tibia	2	1 (100% of fractures—comminuted fracture of proximal end)	2 (50.00% of fractures—comminuted fracture of distal end, 50.00% of fractures—single fracture of proximal end)	28.57	1
Fibula	2	1 (100% of fractures—single fracture of distal end)	1 (100% of fractures—single fracture of proximal end)	28.57	0
Tarsal bones	4	1 (100% of fractures—navicular fracture)	3 (25% of fractures—calcaneus, 25% of fractures—navicular, 25% of fractures—cuboid, 25% of fractures—cuneiform bones)	57.14	0
Metatarsals	2	0	0	28.57	0
Phalanges	0	0	0	0	0

**Table 21:** Fractures in the suicide group.

Damaged Structure	All Cases with Lower Limb Injury	Right	Left	% Damaged Lower Limb	Bilateral
Lower limb	28	17	27	100	16
Femur	16	10 (70.00% of fractures—comminuted fracture of distal end)	12 (33.33% of fractures—single fracture of proximal end, 33.33% of fractures—comminuted fracture of shaft)	57.14	6
Patella	3	2 (100.00% of fractures—comminuted fracture)	2 (50.00% of fractures—single fracture, 50.00% of fractures—comminuted fracture)	10.71	1
Tibia	15	9 (33.33% of fractures—comminuted fracture of distal end, 33.33% of fractures—comminuted fracture of proximal end, 33.33% of fractures—single fracture of proximal end)	13 (38.46% of fractures—comminuted fracture of proximal end)	53.57	7
Fibula	18	9 (44.44% of fractures—comminuted fracture of proximal end)	14 (48.26% of fractures—comminuted fracture of proximal end)	64.29	5
Tarsal bones	10	6 (66.67% of fractures—calcaneus)	9 (55.56% of fractures—calcaneus)	35.71	5
Metatarsals	8	6	6	28.57	4
Phalanges	2	2	0	7.14	0

**Table 22:** Clinical correlations of the strongest statistical findings.

Variable	Associated Fractures	Statistical Value	Clinical Implication
Height of the fall	Fractures of maxilla, mandible, occipital bone (anterior/posterior), temporal bone, lumbar vertebrae, ribs, femur, tibia, fibula	MWU, χ^2^, ANOVA (*p* < 0.01)	Higher fall height correlates with more extensive axial and limb trauma
BMI	Lower BMI → higher frequency of pelvic (pubic, sacral) and forearm fractures	MWU (*p* < 0.05)	Low body mass may predispose to more severe skeletal injury
Manner of death (suicide vs. accident)	Suicides → bilateral fractures (pelvis, limbs, ribs); Accidents → unilateral injuries	χ^2^ (*p* < 0.01)	Bilateral injury distribution may indicate intentional (suicidal) falls
Sex	Females → pubis and sacral fractures	χ^2^ (*p* < 0.05)	Pelvic trauma is more common in female fall victims

## Data Availability

The original contributions presented in this study are included in the article. Further inquiries can be directed to the corresponding authors.

## Abbreviations

The following abbreviations are used in this manuscript:

AI	Artificial Intelligence
APC	Anterior–Posterior Compression
ANOVA	Analysis of Variance
BMI	Body Mass Index
BFT	Blunt Force Trauma
CI	Confidence Interval
DICOM	Digital Imaging and Communications in Medicine
FFF	Free Fatal Falls
LC	Lateral Compression
MWU	Mann–Whitney U test
NAS	Network Attached Storage
OR	Odds Ratio
PAC	Posterior–Anterior Compression
PACS	Picture Archiving and Communication System
PMCT	Post-Mortem Computed Tomography
T-test	Student’s *t*-test
VS	Vertical Shear
